# A Retrospective Study in Trans Individuals Undergoing Gender Affirming Testosterone Treatment: Can Changes in Prolactin Counteract the Negative Effects on the Lipid Profile?

**DOI:** 10.3390/biomedicines13010066

**Published:** 2024-12-30

**Authors:** Jojo Steininger, Katalin Widmann, Ulrike Kaufmann, Marlene Hager, Rodrig Marculescu, Robert Krysiak, Johannes Ott

**Affiliations:** 1 Clinical Division of Gynecologic Endocrinology and Reproductive Medicine, Medical University of Vienna, A-1090 Vienna, Austria; jojo.steininger@meduniwien.ac.at (J.S.); katalin.widmann@meduniwien.ac.at (K.W.); ulrike.kaufmann@meduniwien.ac.at (U.K.); marlene.hager@meduniwien.ac.at (M.H.); 2 Department of Laboratory Medicine, Medical University of Vienna, A-1090 Vienna, Austria; rodrig.marculescu@meduniwien.ac.at; 3 Department of Internal Medicine and Clinical Pharmacology, Medical University of Silesia, 40-055 Katowice, Poland; rkrysiak@sum.edu.pl

**Keywords:** prolactin, HomeoFIT-PRL model, gender-affirming hormone therapy, lipid profile, transgender, testosterone, exogenous sex hormones

## Abstract

**Background/Objectives:** Gender-affirming hormone therapy (GAHT) is known to influence the lipid profiles of trans men and transmasculine individuals. Recent data show that moderate prolactin (PRL) elevations might exert beneficial metabolic effects (“HomeoFIT-PRL model”). The aim of this study is to investigate changes in PRL levels and possible associations between PRL and lipid profiles in this population after a year of GAHT. **Methods:** In a retrospective cohort study, 97 participants, who received GAHT with testosterone, were included. Blood lipids, PRL, and sex steroid hormone levels were evaluated prior to and at 10–14 months after treatment started. **Results:** The difference in PRL levels between baseline and follow-up was significant (*p* = 0.007) with a median difference of +2.3 ng/mL. Concerning blood lipids, the decline in high-density lipoprotein cholesterol (HDL-C) reached statistical significance (median 56 mg/dL versus 50 mg/dL; *p* < 0.001), and low-density lipoprotein cholesterol (LDL-C) and triglyceride levels increased (*p* = 0.023 and *p* = 0.045, respectively). Individuals with a PRL > 25 ng/mL at follow-up (n = 20, 20.6%) revealed increases in total cholesterol and LDL-C significantly less often. Overall, participants frequently displayed unfavorable changes in their lipid profile after 10–14 months of GAHT, as well as a slight but significant increase in PRL. About 20% of patients showed mild-to-moderate hyperprolactinemia (PRL > 25 ng/mL). However, such changes were associated with potentially beneficial dynamics in the lipid profile, at least for triglycerides. **Conclusions:** These findings seem in line with the HomeoFIT-PRL model suggesting that moderate elevations in PRL levels might exert beneficial metabolic effects. Increases in PRL after testosterone were common.

## 1. Introduction

In recent years, it has become clear that prolactin (PRL) exerts relevant favorable effects on metabolic homeostasis [1]. In humans, low PRL levels have been associated with an increased prevalence of various metabolic diseases [1,2], whereas overweight and obese patients, who showed increased PRL levels, also revealed better metabolic profiles than BMI-matched patients with lower PRL values [1,3,4,5,6,7]. Notably, after normalization of low PRL levels, the metabolic profile, disturbed at baseline, did not differ from that observed in drug-naïve, apparently healthy young women [8]. However, an excess in PRL promotes metabolic syndrome and impairs lipid profiles [9]. The HomeoFIT-PRL model, described by Macotela et al. proposes that moderately elevated PRL levels, in the upper normal physiological range and slightly above it, are associated with metabolic fitness and homeostasis [1]. The model links PRL levels with reduced insulin resistance, improved glucose tolerance, decreased adipose tissue hypertrophy, and a protective effect against metabolic syndrome and diabetes type two development. As such, is it part of the concept of homeorhetic response, which implicates PRL levels as an adaptive mechanism to metabolic challenges, both physiological and pathological [1].

Of note, when focusing on dyslipidemia, inverse associations were found between slightly elevated PRL levels and total cholesterol, low-density lipoprotein cholesterol (LDL-C), and triglyceride levels [5,6,10,11]. However, longitudinal data on metabolic status and accompanying PRL levels are scarce. One small study about the course of PRL in prediabetic patients with polycystic ovary syndrome (PCOS) and hyperprolactinemia showed that metformin led to an improvement in insulin resistance, which was not accompanied by a decrease in PRL. In contrast, the non-PCOS group with hyperprolactinemia revealed both a decrease in the homeostatic model assessment of insulin resistance and PRL, suggesting that testosterone impaired the PRL decrease [12].

Thus, it would be of interest to see the course in PRL and its implications in terms of metabolic changes. Only a few patient groups allow for a planned observation of gross metabolic changes. One of these groups is trans men and transmasculine people—individuals who were assigned female at birth (AFAB) but identify as men or masculine individuals—who receive gender-affirming testosterone therapy (GAHT), which (amongst other effects) impacts metabolic biomarkers. Notably, alterations in the lipid profiles are frequently observed in these patients, which include decreases in high-density lipoprotein cholesterol (HDL-C) and increases in LDL-C [13,14]. According to a recent meta-analysis, all fractions of the lipid profile are negatively affected, which outlines the potential side effects of testosterone-containing GAHT [15]. Usually, these patients also undergo regular follow-up examinations of various hormone levels. Thus, we aimed to retrospectively analyze our population of these patients and to focus on changes in PRL levels and their association with total cholesterol and triglycerides within the first ten to fourteen months of testosterone treatment.

## 2. Materials and Methods

### 2.1. Patient Population

**Figure 1:**
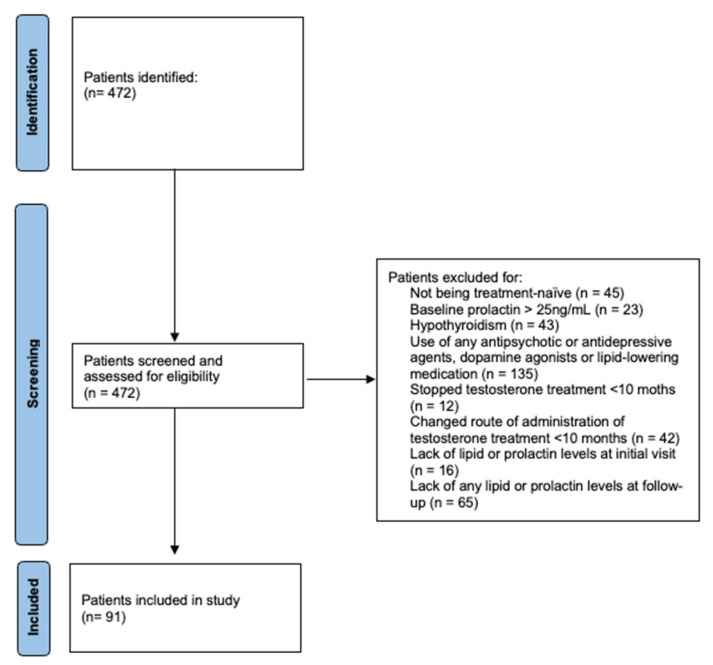
PRISMA flow chart of patient identification, screening, and inclusion.

In a retrospective cohort study, we included 97 AFAB individuals, who started GAHT with testosterone at the Clinical Division of Gynecologic Endocrinology and Reproductive Medicine of the Medical University Vienna, Vienna, Austria, from January 2013 to September 2023. All individuals met the following inclusion criteria: AFAB individuals undergoing standard GAHT (see below) with testosterone for at least ten months; initiation of testosterone treatment after the initial visit at our department (all of the patients had to be treatment-naïve and thus presenting with sex hormone levels within the standard range for female individuals prior to treatment); patients who underwent a follow-up visit after ten to fourteen months after treatment initiation (during the first year, patients have to follow up-appointments every 8–16 weeks; thus, this timeframe was chosen to include as many patients as possible around the 1 year mark); patients who did not undergo any change in GAHT regimen during the study period; and patients who did not take any lipid-lowering medication at the time of treatment initiation. The following exclusion criteria were applied: baseline PRL > 25 ng/mL (hyperprolactinemia), pregnancy, lactation, hypothyroidism (treated and untreated), use of any antipsychotic or antidepressive agents, use of dopamine agonists, previous radiotherapy, pituitary tumors, hypophysitis, and neurosurgical interventions (see Figure 1). Diagnosis and the indication for somatic treatment were thoroughly made by psychiatrists and supported by psychotherapists and clinical psychologists according to the standards of care of WPATH [16]. All procedures were carried out according to the Good Scientific Practice Guidelines set forth by the Medical University of Vienna, which are based on the Helsinki Declaration.

### 2.2. Standard Gender Affirming Hormone Therapy (GAHT) with Testosterone

The standard GAHT for trans men and transmasculine individuals included intramuscular testosterone undecanoate (1000 mg every 8–14 weeks, Nebido^®^: Bayer AG, Leverkusen, Germany) or transdermal formulations (testosterone gel (Testogel: Besins Healthcare Germany GmbH, Berlin, Germany or Testavan Gel: The Simple Pharma Company Limited, Dublin, Ireland) or a testosterone cream (magistral formulation), applied daily. The starting dose of transdermal applications was 50 mg/day regardless of formulation. During the follow-ups within the next 12 months (every 8–14 weeks), doses were adjusted depending on testosterone levels to reach values within the male normal range. After 12 months, doses varied from 40 mg to 200 mg, depending on the individual. Daily oral administration of 75 µg desogestrel (Moniq Gyneal Mono: milbe GmbH, Brehna, Germany) or lynestrenol 5 mg (Orgametril: Organon Healthcare GmbH, München, Germany) was also provided frequently, in case the patients wanted to become amenorrhoeic as soon as possible. Few patients received GnRH-analogues triptorelin (1 mg/day, subcutaneously, Decapeptyl: Ferring GmbH, Saint-Prex, Swizerland) or leuprorelin acetate (3.75 mg/month, intramuscularly, Enantone: Takeda GmbH, Konstanz, Germany) at their wish and after individual counseling in order to attain amenorrhea as fast as possible and suppress endogenous sex steroid production. All patients were routinely advised to stop smoking and, if overweight, to lose weight and improve their diet to improve cardiovascular health.

### 2.3. Parameters Analyzed

Blood samples were obtained from a peripheral vein after at least 10 h overnight fasting period on cycle days 2–5 before the treatment and also after treatment if possible and were analyzed at the local ISO-certified Department of Laboratory Medicine, General Hospital of Vienna, Vienna, Austria, according to ISO 15189 quality standards. Worth noting, blood samples were drawn after at least 30 min resting in a seated position. All parameters were routinely assessed before (baseline) and at ten to fourteen months after treatment initiation (follow-up). Hormone determinations were performed by electrochemiluminescence immunoassays (ECLIAs) on the Cobas 8000 fully automated clinical laboratory platform (Roche Diagnostics, Mannheim, Germany). The inter- and intra-assay coefficients of variation (%) and limits of detection of the individual assays were, respectively, 4.4, 3.1 and 0.094 ng/mL (2 µIU/mL) for PRL, 12.3, 8.4 and 5 pg/mL (18.4 pmol/L) for estradiol, 11.5, 5.7, and 0.025 ng/mL (0.087 nmol/L) for testosterone, 3.9, 2.1, and 0.3 mIU/mL for FSH, 2.3, 2.2, and 0.3 mIU/mL for LH, 13.8, 11.2, and 0.2 µg/dL (0.005 µmol/L) for DHEAS, and 3.5, 3.1, and 0.8 nmol/L for SHBG.

The main outcome parameter was total PRL levels. Other baseline and follow-up laboratory parameters included total cholesterol, HDL-C, LDL-C, and triglycerides. Total cholesterol, triglycerides, LDL-C, and HDL-C were measured using the corresponding colorimetric enzymatic Cobas assays (CHOL2, TRIGL, LDLC3, and HDLC4, respectively) on the Cobas 8000 platform (Cobas c 702 modules, oche Diagnostics, Mannheim, Germany). Concerning endocrine function besides PRL, the focus was on estradiol, testosterone, follicle-stimulating hormone (FSH), luteinizing hormone (LH), testosterone, dehydroepiandrosterone sulfate (DHEAS), and sex hormone-binding globulin (SHBG), all measured by the corresponding Cobas electrochemiluminescence immunoassays (ECLIAs) on Cobas e 602 analyzers (Roche, Mannheim, Germany). We also included hemoglobin, erythrocyte count, hematocrit, and transaminases GOT and GPT. Complete blood counts were generated with Sysmex XN hematology analyzers (Sysmex, Kobe, Japan). GOT and GPT were measured by the IFCC-conform Cobas ASTPM and ALTPM enzyme activity assays with pyridoxal phosphate activation, respectively, on the Cobas 8000 clinical chemistry platform (Cobas c 702 modules, Roche, Penzberg, Germany). Finally, basic patient characteristics were recorded, which included age at treatment initiation, body mass index (BMI), smoking status, and the type of GAHT. All data were retrieved using the AKIM software (IS-H release 618, support package 0025) of the General Hospital Vienna.

### 2.4. Statistical Analysis

Variables are described by frequencies and median (interquartile ranges, IQR). Paired *t*-tests were used to test for differences between initial and follow-up parameters. Normal distribution of the metric variables was verified before using a *t*-test. Univariate correlations between some variables were sought by Pearson and Spearman tests. To evaluate possibly associated factors with categorical data, univariable binary regression models were used. All significant parameters were then entered into a multivariable binary regression model. For these models, odds ratios (OR), their 95% confidence intervals (95% CI), and *p*-values are provided. For calculation of an optimized cut-off value, receiver operator characteristic (ROC) curves were used. The cut-off with the maximum sum of sensitivity and specificity was defined as the optimized cut-off value. *p*-values < 0.05 were considered statistically significant. Statistical analysis was performed in SPSS 28.0.1.0 for Windows (SPSS Inc., Chicago, IL, USA, 1989–2023).

## 3. Results

At the time of treatment initiation, the median age was 23 years (IQR 20–28). Twenty-nine individuals (29.9%) were smokers. Thirty-one (32.0%) patients received testosterone undecanoate, whereas sixty-six individuals (68.0%) were treated with transdermal testosterone. Notably, 54 (55.7%) individuals received testosterone treatment only. In the remaining 43 cases, daily oral desogestrel and lynestrenol were provided in 34 (35.1%) and 5 (5.2%) patients, respectively. A GnRH agonist (triptorelin or leuprolin acetate) was given to six (6.2%) individuals. Notably, one patient received both desogestrel and a GnRH agonist in addition to testosterone therapy. Table 1 provides details about patient characteristics and laboratory measurements at baseline and at 10–14 months follow-up. PRL levels increased during this time span (median 15.2 ng/mL versus 17.5 ng/mL; *p* = 0.007). BMI, erythrocyte count, hemoglobin, and hematocrit increased significantly (*p* < 0.001) during that timeframe, while liver enzymes remained unchanged. LH and FSH decreased significantly under GAHT (*p* < 0.001 and *p* = 0.035, respectively).

At baseline, there were no significant correlations between PRL levels and the lipid profiles (PRL versus total cholesterol: Pearson r = −0.153, *p* = 0.135, Spearman r = −0.127, *p* = 0.213; PRL versus LDL-C: Pearson r = −0.183, *p* = 0.074, Spearman r = −0.160, *p* = 0.118; PRL versus HDL-C: Pearson r = 0.049, *p* = 0.638, Spearman r = 0.057, *p* = 0.581; PRL versus triglycerides: Pearson r = −0.100, *p* = 0.330, Spearman r = −0.051, *p* = 0.622).

**Figure 2:**
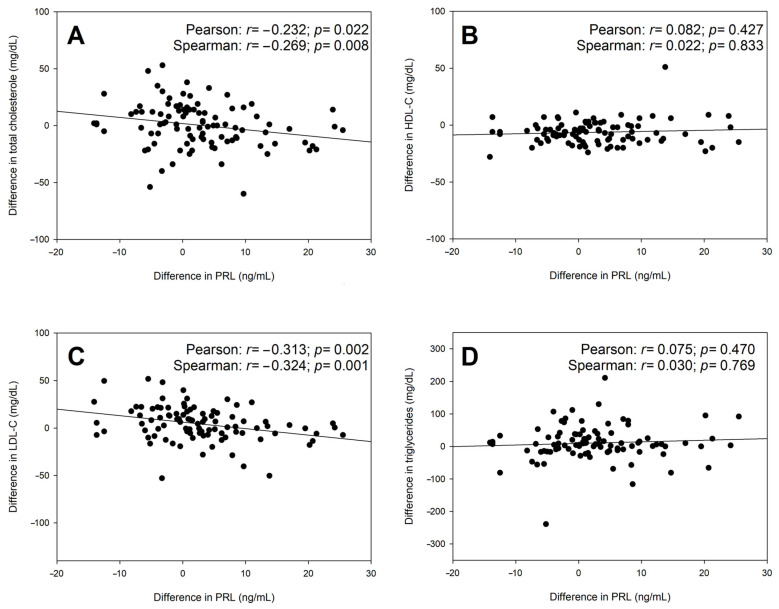
Differences in PRL and lipid levels between baseline and follow-up examinations: correlation analyses. (**A**) correlation between difference in total colesterole and prolactin (PRL), (**B**) correlation between difference in HDL-C and PRL, (**C**) correlation between difference in LDL-C and PRL, (**D**) correlation between difference in triglycerides and PRL.

In the paired *t*-test, the difference in PRL levels between the baseline and follow-up was significant (*p* = 0.007, Table 1; median difference: 1.2 ng/mL, IQR: −3.1;7.1). Concerning lipid values, there were significant increases in triglyceride (median 70 mg/dL versus median 81 mg/dL, *p* = 0.045) and LDL-C levels (median 85.6 mg/dL versus 91.0 mg/dL, *p* = 0.023) and a significant decline in HDL-C (median 56 mg/dL versus 50 mg/dL; *p* < 0.001; Table 1). In the next step, we focused on the differences in serum levels of PRL and lipids between the baseline and follow-up. The differences in PRL levels were correlated to the differences in lipid levels (Figure 2). Significant (negative) correlations were only found between PRL and total cholesterol as well as PRL and LDL-C (*p* < 0.05). However, from baseline to ten- to fourteen-months follow-up, increases in total cholesterol, LDL-C, and triglycerides were found in 49 (50.5%), 55 (56.7%), and 61 (62.9%), respectively, whereas HDL-C decreased in 73 (75.3%) individuals. When focusing on PRL and testosterone/estradiol dynamics, there were no significant correlations (PRL versus testosterone: Pearson r = −0.110, *p* = 0.292, Spearman r = −0.096, *p* = 0.359; PRL versus estradiol: Pearson r = 0.151, *p* = 0.146, Spearman r = 0.127, *p* = 0.222).

According to the HomeoFIT-PRL (Homeostatic Functionally Increased Transient Prolactinemia) model, mild hyperprolactinemia with PRL levels > 25 ng/mL has been claimed to occur in response to metabolic challenges and to favor metabolic homeostasis [1,2]. Since a baseline PRL > 25 ng/mL had been an exclusion criterion, we were able to focus on PRL > 25 ng/mL at follow-up, which was found in 19 patients (19.6%). Patients were subdivided accordingly (Table 2). It is important to note that none of these patients had a PRL > 47 ng/mL and can thus all be classified as mild-to-moderate hyperprolactinemia. Notably, in univariable logistic regression analyses, patients with a PRL > 25 ng/mL at follow-up revealed a decrease in triglyceride levels (median difference from baseline to follow-up: 9 mg/dL, IQR −1–85 versus 7 mg/dL, IQR −16–29; *p* = 0.024). Since this was the only statistically significant parameter in the univariable model, the multivariable model revealed the same result.

**Figure 3:**
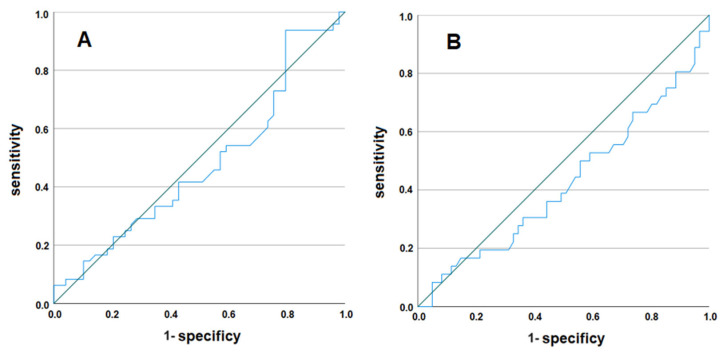
ROC analysis for the optimized cut-off for follow-up PRL levels. Patients were subdivided into those who revealed a stable course or a decline in total cholesterol (**A**) and triglyceride (**B**) levels and those who experienced an increase at follow-up.

Finally, we evaluated the optimized cut-off level for PRL at follow-up (Figure 3). First, patients were subdivided into those who revealed a stable course or decline in total cholesterol levels and those who experienced an increase at follow-up. The maximum sum of sensitivity and specificity was reached at a follow-up PRL level of 15.1 ng/mL (sensitivity = 67.3%, specificity = 45.8%; *p* = 0.131). Then, patients were subdivided into those with a stable course or a decline in triglyceride levels versus those with an increase. In this analysis, the maximum sum of sensitivity and specificity was reached at a follow-up PRL level of 14.8 ng/mL (sensitivity = 70.5%, specificity = 44.4%; *p* = 0.103).

## 4. Discussion

Our retrospective study confirms previous observations that many trans men and transmasculine individuals, who receive GAHT, reveal worsening lipid profiles (increases in total cholesterol, LDL-C, and triglycerides in 50.5–62.9% after 10–14 months of GAHT) [14]. This was reflected by the fact that there were significant increases in total cholesterol and LDL-C as well as a decrease in HDL-C. After the observation period of 10–14 months of testosterone treatment, about 20% of the patients revealed elevated PRL levels > 25 ng/mL (hyperprolactinemia), which have been claimed to exert beneficial metabolic effects according to the HomeoFIT-PRL model [1,2]. However, our results are not in line with previous observations since these individuals revealed a statistically higher decrease in triglyceride levels (Table 2) but no associations with the dynamics of other lipid levels. Notably, the optimized PRL cut-off values at follow-up were around 15 ng/mL in our data set when changes in total cholesterol and triglycerides were used as the reference parameters. When focusing on previous data about PRL and dyslipidemia, inverse associations were found between moderately elevated PRL levels and total cholesterol, LDL-C, and triglyceride levels in cross-sectional studies [5,6,10,11]. This is in accordance with our findings since, in our data set, increases in PRL levels from baseline to follow-up were negatively correlated with the changes in total cholesterol and LDL-C levels in the same time frame (Figure 3). It remains unclear, why there were no significant associations between PRL and HDL-C or triglycerides. However, to the best of our knowledge, we present the first longitudinal data about changes in PRL levels in a patient population that is at risk of developing unfavorable alterations in their lipid profiles and has been shown to have an increased cardiovascular mortality risk [17,18]. Again, it turns out that moderate increases in PRL seem to be metabolically protective. However, according to our data, the dynamics in PRL levels seem to be of higher relevance than absolute PRL levels at follow-up.

Despite these reassuring findings, it needs to be stated that there were no significant associations between PRL levels and the lipid profile at baseline, although one could argue that there was a trend towards a negative correlation between PRL and LDL-C in the Pearson test (r = −0.183, *p* = 0.074). This could be due to the fact that the majority of our patients revealed a normal lipid profile at baseline (Table 1), contrary to the populations of most other studies investigating PRL and lipid profiles [3,4,5,6,7,10,11,12].

A GnRH-stimulated PRL release, which might be mediated by a paracrine FSH effect, has been reported [19]. Recently, it was shown that a decline in hypothalamic GnRH pulsatility was associated with a decline in PRL levels [20]. Thus, downregulation of the hypothalamic–pituitary–ovarian axis with GnRH-analogues should lead to a decrease in PRL levels, which was the case in one of six patients on this treatment.If these patients did not have the capacity to increase their PRL, this could imply that the concomitant use of GnRH-analogues with testosterone treatment might be associated with an increased metabolic risk. However, to the best of our knowledge, this has not been evaluated so far.

While this study is, to our knowledge, the first study to ever investigate the role of PRL in metabolic changes occurring under testosterone-based GAHT and adds valuable data to the HomeFIT PRL model, several limitations have to be addressed: Due to the retrospective design of this study, several factors that potentially influence PRL levels, such as lifestyle, sports, and stress levels, could not be assessed. Since a healthy lifestyle, which included a healthy diet and sportive activity, was recommended to all individuals before the start of testosterone treatment, several patients might have experienced an improvement in the lipid profile. Empirically, many AFAB individuals who start their gender-affirming testosterone treatment also start training to build muscle and thus achieve a more masculine physique quickly. It must be noted that regular exercise might increase PRL levels, although conflicting results have been published, as reviewed recently [21]. While all patients received information and recommendations regarding lifestyle and were recommended regular exercise and a healthy diet, unfortunately, our retrospective data set does not include information about dietary and lifestyle habits as well as on changes in body composition, which must be addressed as a study limitation. Another limitation is the relatively short follow-up period. Longer observational periods would be beneficial to investigate whether the moderate PRL increase in AFAB individuals under GAHT is transient or permanent and whether it is associated with favorable long-term changes to lipid profiles, which are known to be worsening under testosterone GAHT [14,15] More research with a longer follow-up period is necessary to shed light on the correlations we observed. Furthermore, it might be of interest to assess stress levels, as well as the physical activity of patients when investigating PRL levels, as these stimuli can also lead to an increase in PRL and could be a potential confounder [9]. All in all, one cannot be entirely sure which changes have led to the dynamics in PRL. Theoretically, both the changing testosterone levels and the aforementioned changes in the lipid profile as well as the unevaluated lifestyle modifications mentioned could be responsible. Future prospective studies will have to shed light on the question of causality. Notably, it has been outlined in guidelines and recommendations that GAHT with estrogens and/or testosterone-lowering progestogens would lead to drug-induced hyperprolactinemia. No such data are known for GAHT with testosterone [15,22,23]. Although we cannot completely rule out a PRL-increasing effect of testosterone, we chose not to use the term “drug-induced hyperprolactinemia”. Despite the lack of knowledge about the exact causes for changes in PRL levels, which might be considered a minor study limitation, we believe that our data still allowed us to assess associations between PRL and the lipid profile.

Additionally, further studies in transmasculine populations should evaluate chest-binding practices. The effects of chest binding on PRL, a common practice amongst trans men and transmasculine individuals prior to chest masculinization surgery, are yet to be determined—but chest wall stimulation as well as nipple stimulation might increase PRL levels [24]. As chest binding practices were not assessed in detail, this can be seen as an additional study limitation. Only one individual underwent chest masculinization surgery during the follow-up period.

It must be mentioned that it was not the aim of this study to assess the side effects or potential risks of GAHT but to investigate the known side effects of worsening lipid profiles [14,15] through the lens of the HomeoFIT model. Last but not least, the retrospective study design in itself and the sample size must also be seen as study limitations.

## 5. Conclusions

In conclusion, trans men and transmasculine individuals, who received testosterone in the course of GAHT, revealed unfavorable changes in their lipid profile frequently as well as a minor but significant increase in PRL levels. From baseline to a follow-up visit after ten to fourteen months, increases in PRL were associated with more favorable courses of total cholesterol and LDL-C. This is the first longitudinal study on changes in PRL and the lipid profile in individuals at risk, which confirms that PRL levels > 25 ng/mL might be metabolically protective. Larger, prospective studies are warranted to prove our findings.

## Figures and Tables

**Table 1:** Patient characteristics and results of laboratory analyses at baseline and at 10–14 months follow-up. Data are presented as median (IQR) for numerical parameters or n (frequency) for categorical parameters; abbreviations: BMI, body mass index; GOT, Aspartate transaminase; GPT, Alanine transaminase; HDL-C, high-density lipoprotein cholesterol; LDL-C, low-density lipoprotein cholesterol; TSH, thyrotropin; FSH, follicle-stimulating hormone; LH, luteinizing hormone, SHBG, sexual hormone binding globulin; PRL, prolactin.

	Baseline	10–14 Months Follow-Up	*p* (Paired *t*-Test)
BMI (kg/m^2^)	22.6 (20.5;26.8)	23.2 (20.7;26.8)	<0.001
Erythrocyte count (T/L)	4.6 (4.4;4.9)	5.1 (4.9;5.4)	<0.001
Hemoglobin (g/dL)	13.5 (12.8;14.3)	14.9 (14.3;15.7)	<0.001
Hematocrit (%)	40.0 (38.2;41.9)	44.0 (42.4;46.3)	<0.001
GOT (U/L)	19 (16;23)	20 (17;24)	0.223
GPT (U/L)	17 (13;22)	19 (15;24)	0.053
Triglycerides (mg/dL)	70 (53;107)	81 (56;126)	0.045
Total cholesterol (mg/dL)	159 (140;181)	156 (143;189)	0.849
HDL-C (mg/dL)	56 (47;67)	50 (41;60)	<0.001
LDL-C (mg/dL)	85.6 (68.5;103.8)	91.0 (70.8;111.5)	0.023
TSH (µIU/mL)	1.77 (1.19;2.40)	2.05 (1.32;2.69)	0.091
FSH (mIU/mL)	5.3 (3.9;6.5)	4.9 (3.1;6.1)	0.035
LH (mIU/mL)	9.1 (6.2;12.2)	5.2 (2.2;9.7)	<0.001
Estradiol (ng/mL)	70 (40;116)	50 (33;64)	<0.001
Testosterone (ng/mL)	0.36 (0.27;0.51)	4.92 (3.97;6.86)	<0.001
SHBG (nmol/L)	56.6 (31.4;68.4)	27.9 (20.5;38.3)	<0.001
PRL (ng/mL)	15.2 (11.4;21.4)	17.5 (12.5;23.4)	0.007

**Table 2:** Comparison of individuals with and without PRL > 25 ng/mL at 10–14-months follow-up. Binary logistic regression model evaluating associated parameters. Data are presented as * median (IQR) for numerical parameters or # number (frequency) for categorical parameters.

	Follow-Up PRL		OR (95%CI)	*p*	Adj. OR (95%CI)	*p*
	>25 ng/mL (n = 19)	≤25 ng/mL (n = 78)				
Age (years) *	23 (19;28)	22 (20;28)	0.991 (0.910;1.078)	0.833	-	-
Smoking #	4 (21.1)	25 (32.1)	0.565 (0.170;1.879)	0.352		
Intramuscular testosterone #	4 (21.1)	27 (34.6)	0.504 (0.152;1.668)	0.262	-	-
Daily oral desogestrel #	6 (31.6)	28 (35.6)	0.824 (0.282;2.408)	0.724	-	-
Daily oral lynestrenol #	0	5 (6.4)	0 (0;-)	1.000	-	-
GnRHa treatment #	1 (5.3)	5 (6.4)	0.811 (0.089;7.380)	0.853	-	-
Difference in BMI (kg/m^2^) *	0.6 (−0.2;1.2)	0.4 (−0;0.7)	1.324 (0.707;2.478)	0.381	-	-
Difference in total cholesterol (mg/dL) *	8 (−10;24)	1 (−15;12)	1.025 (0.997;1.054)	0.078	-	-
Increase in total cholesterol #	10 (52.6)	39 (50.0)	1.111 (0.407;3.032)	0.837	-	-
Difference in HDL-C (mg/dL) *	−7 (−15;2)	−6 (−13;−1)	0.989 (0.940;1.041)	0.680	-	-
Decline in HDL-C #	14 (73.7)	59 (75.6)	0.902 (0.287;2.832)	0.859	-	-
Difference in LDL-C (mg/dL) *	4.8 (−2.2;12.8)	4.4 (−7.3;16.5)	1.013 (0.985;1.042)	0.367	-	-
Increase in LDL-C #	12 (63.2)	43 (55.1)	1.395 (0.496;3.922)	0.528	-	-
Difference in triglycerides (mg/dL) *	9 (−1;85)	7 (−16;29)	1.013 (1.002;1.024)	0.024	1.013 (1.002;1.024)	0.024
Increase in triglycerides #	13 (68.4)	48 (61.5)	1.354 (0.465;3.946)	0.578	-	-

## Data Availability

The data presented in this study are available upon request from the corresponding author due to privacy reasons.
